# The use of systematic reviews for conducting new studies in physiotherapy research: a meta-research study comparing author guidelines of physiotherapy-related journals

**DOI:** 10.1186/s13643-023-02427-7

**Published:** 2024-01-13

**Authors:** Diane Rosen, Nils L. Reiter, Barbara Vogel, Robert Prill

**Affiliations:** 1 Centre of Evidence-Based Practice in Brandenburg: A JBI Affiliated Group, Brandenburg Medical School Theodor Fontane, Brandenburg a.d.H., Germany; 2 Berlin School of Public Health, Berlin, Germany; 3 Alice Salomon University of Applied Sciences Berlin, Berlin, Germany; 4 Department of Orthopaedics and Sports Orthopaedics, Physical Therapy, Klinikum Rechts Der Isar, Technical University Munich, Munich, Germany; 5 Center of Orthopaedics and Traumatology, University Hospital Brandenburg/Havel, Brandenburg Medical School Theodor Fontane, Brandenburg a.d.H, Germany; 6 Faculty of Health Sciences Brandenburg, Brandenburg Medical School Theodor Fontane, Brandenburg a.d.H, Germany

**Keywords:** Systematic review, Evidence-based research (EBR), Physiotherapy, Physical therapy, Scientific medical journals, Publication guidelines, Author guidelines, Evidence-based practice (EBP), Meta research

## Abstract

**Background:**

Requiring authors to base their research on a systematic review of the existing literature prevents the generation of redundant scientific studies, thereby avoiding the deprivation of effective therapies for trial participants and the waste of research funds. Scientific medical journals could require this in their author guidelines. While this applies to all areas of research, it is also relevant to physiotherapy and rehabilitation research, which predominantly involve interventional trials in patients.

**Objective:**

The aim of this study was to determine the extent to which the use of systematic reviews to justify a new trial is already being requested by physiotherapy-related scientific medical journals (PTJs). In addition, a comparison was made between PTJs and scientific medical journals with the highest impact factor in the Science Citation Index Extended (SCIE).

**Methods:**

This meta-research study is based on a systematic examination of the author guidelines of 149 PTJs. The journals were identified and included based on the number of publications with physiotherapy as a keyword in the databases PEDro, and Medline (Pubmed). The included author guidelines were analysed for the extent to which they specified that a new trial should be justified by a systematic review of the literature. Additionally, they were compared with 14 scientific medical journals with the highest impact factor in the SCIE (LJs).

**Results:**

In their author guidelines, none of the included PTJs required or recommended the use of a systematic review to justify a new trial. Among LJs, four journals (28.57%), all associated with the Lancet group, required the study justification through a systematic review of the literature.

**Conclusion:**

Neither PTJs nor LJs require or recommend the use of a systematic review to justify a new trial in their author guidelines. This potentially leaves room for unethical scientific practices and should be critically considered in future research.

**Supplementary Information:**

The online version contains supplementary material available at 10.1186/s13643-023-02427-7.

## Background

The number of published scientific papers is constantly increasing [1, 2]. Consequently, it is becoming more challenging for end-users to distinguish between high-quality, relevant literature, and research waste which is redundant and irrelevant [3]. Furthermore, conducting redundant research is unethical and wastes human and financial resources [4]. In the field of physiotherapy research, redundant studies are still being carried out. One example is research into the effects of exercise in patients with knee osteoarthritis. Although previous trials and a series of Cochrane reviews have shown and summarised the benefits of exercise [5–7], research on a similar topic is still ongoing [8].

To counteract research waste and to conduct research in a transparent manner, the Evidence-Based Research (EBR) approach was developed [9, 10]. It states that new research should be justified by a systematic review (SR) of the existing literature and that the results of a new study should be discussed in the context of a SR [9]. According to the EBR Network a SR is defined as “a structured and preplanned synthesis of original studies that consists of predefined research questions, inclusion criteria, search methods, selection procedures, quality assessment, data extraction, and data analysis” [11]. The EBR approach has been shown to be able to reduce research waste to some extent [12]. However, its use in general medicine [13–17] or physiotherapy and rehabilitation research is questionable [18].

Among others, scientific medical journals are relevant stakeholders in implementing the EBR approach [9, 18] as they play an important role in avoiding research waste [19, 20]. Scientific medical journals could for instance require their authors to justify new trials through SRs. Such recommendations apply to general medicine but are also relevant to physiotherapy. Most physiotherapy research is based on randomised controlled trials (RCTs) involving patients [18], some of whom receive unnecessary treatment if previous trials have already shown the effects of a particular intervention and there is no question of clinical equipoise.

According to Ioannidis, meta-research is the study of research itself: its methods, reporting, reproducibility, evaluation and incentives [21]. The aim of this meta-research study was to determine the extent to which physiotherapy-related scientific medical journals (PTJs) require the use of SRs for the study rationale in their author guidelines for RCTs and compare them with the author guidelines of scientific medical journals with a high impact factor in the Science Citation Index Extended (SCIE), hereafter referred to as leading journals (LJs). The comparison with LJs places the publication practices of PTJs in the current context of academic methodology, modeled on journals with potentially the highest scientific quality and relevance to their specific field. It was hypothesised that PTJs would be less likely to require the use of SRs to justify a new trial than LJs.

## Methods

In this meta-research study, i.e. study on research practice, author guidelines for RCTs of PTJs and LJs were systematically reviewed. PTJs were defined as journals publishing RCTs relevant to physiotherapy. To identify PTJs, three strategies were combined.

As a first strategy, the PEDro database [22], which specialises in the publication of research relevant to physiotherapy, was accessed. All clinical trials referenced and published in the last five years (between January 2016 and August 2021) were extracted and organised by the journal in which they were published using an automated Microsoft Excel (version 16.70) macro tool. Journals were then ranked by the number of publications and included if at least 22 publications, including one RCT, were referenced. The cut-off was set at 22 publications because journals were only considered relevant to physiotherapy if at least one physiotherapy-related study was published every three months. As no standard procedure on this behalf is known to the authors, this cut-off was also determined for feasibility reasons.

As a second strategy, the Medline database [23] was searched via PubMed using the terms: ‘physiotherapy’ OR ‘physical therapy’ and the filters ‘humans’, ‘clinical trial’, and ‘RCT’. Studies were extracted and organised using the aforementioned Excel macro tool and journals were included using the same cut-off of 22 physiotherapy-related studies.

Furthermore, another list was compiled of all journals in the rehabilitation category of the SCIE database [24], with an impact factor of at least 1.0. regardless of their number of publications in PEDro [22] or PubMed [23]. Journals in the categories ‘Sports Science’ and ‘Orthopaedics’ which are closely related to physiotherapy and rehabilitation research, were added to this list if at least 10 publications, were referenced in PEDro [22] or PubMed [23] according to the above criteria. The cut-off was set lower for these three categories as they were considered relevant to physiotherapy research but not expected to publish only studies with physiotherapy as a keyword.

All included journals were combined into a final list and duplicates were removed. The final list was then compared with 25 LJs. These were defined as the 25 journals with the highest impact factor in the Journal Citation Reports (JCR) in the SCIE and therefore can be considered the most influential journals in medical science. The filter ‘Clinical Medicine’ was applied in order to exclude scientific medical journals that are concerned with basic sciences.

A data extraction protocol was used in accordance with the EBR criteria to systematically search the websites of the journals. One researcher (DR) identified their publication guidelines by searching the homepage using the following search terms: ‘author guidelines’, ‘information for authors’, ‘submission guidelines’, ‘reporting guidelines’, ‘submission checklist’, ‘for authors’, and ‘about’. In the identified section, it was determined whether journals required the use of SRs to justify a new study, whether appropriate background was required, whether no background was mentioned, or whether the journal explicitly stated that no systematic review of the literature was needed. In case of uncertainty, a second researcher (RP) verified the findings.

Additionally, it was assessed if journals referred to other reporting standards that require an appropriate background. These were referrals to the Enhancing the Quality and Transparency of Health Research (EQUATOR) Network, the International Committee of Medical Journal Editors (ICMJE), the Consolidated Standards of Reporting Trials (CONSORT), or the Standard Protocol Items: Recommendations for Interventional Trials (SPIRIT) checklists [25–28].

The following terms were searched: ‘background’, ‘rationale’, ‘intro’, ‘reporting’, ‘systematic review’, ‘consort’, ‘spirit’, ‘equator’, ‘icmje’, ‘international’. The results for the two groups, PTJs, and LJs, were then compared.

## Results

According to the search strategy, 12,233 clinical trials which were published in 2024 journals were identified via the PEDro database and 21,197 clinical trials which were published in 1,994 journals were identified via PubMed. With the cut-off set at 22 publications, 112 journals were identified via the PEDro database, and 101 journals were identified via PubMed. According to the inclusion criteria, the search of the SCIE database resulted in 42 extracted PTJs. After removing duplicates, the final list contained 152 PTJs.

**Fig. 1:**
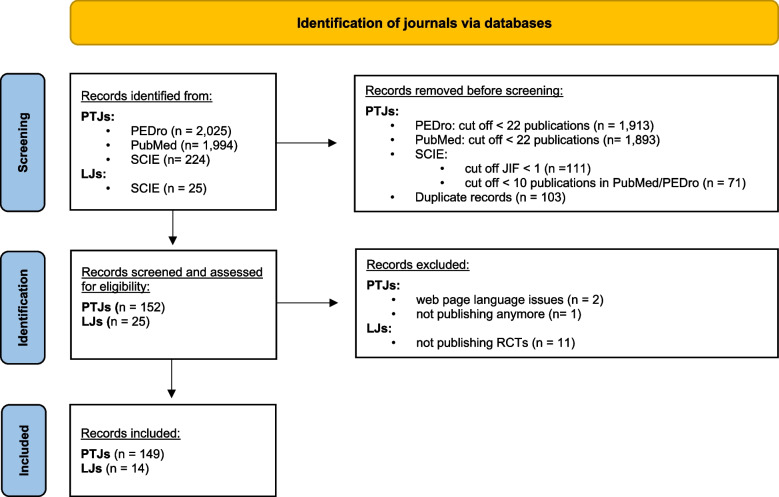
Flow chart for the journal selection process

Three PTJs were excluded from the analysis. One because it was not actively publishing anymore, and two because they published in languages other than German or English. Eleven of the 25 LJs were excluded as they did not publish RCTs. Therefore, after exclusion, in total, 149 PTJs were compared with 14 LJs. PTJs cover a broad range of journals, varying in impact factor (from 0.519 to 9.139) as well as in the SCIE category with most journals belonging to the category ‘Orthopedics’ (*n* = 18). The full list can be found in Additional file 1. The journal selection process is illustrated in Fig. 1.

None of the PTJs required an SR for the study rationale in their author guidelines. Four of the LJs (28.57%), all associated with The Lancet group [29], required a prior SR of the literature. The journals are: 'The Lancet’, ‘Lancet Oncology’, ‘Lancet Diabetes & Endocrinology’, as well as ‘Lancet Respiratory Medicine’. As shown in Table 1, an appropriate background was required in 20.13% of the PTJs and 21.43% of the LJs. These included journals within the SAGE publishing group [30] and Frontiers journals, which implemented a specific guideline known as VALID, where ‘D’ represents the requirement for grounding studies in existing literature through sufficient referencing and appropriate coverage of relevant literature [31]. The majority, 79.87% of PTJs and 50% of all LJs examined, either did not require a literature-based background or explicitly discouraged authors from using SRs to provide a rationale for the study. No specific reason was given by these journals, most of which are part of the Elsevier publishing group [32]. Most of the PTJs (74.50%) and the LJs (92.86%) refer to the reporting standards CONSORT or SPIRIT.

**Table 1:** Results

Category		PTJs *n*(%)	LJs *n*(%)	Overall *n*(%)
		149 (100)	14 (100)	163 (100)
SR in rationale	SR in rationale is required	0 (0)	4 (28.57)	4 (2.45)
	Appropriate background needed	30 (20,13)	3 (21.43)	33 (20.25)
	State nothing regarding background	103 (69,13)	6 (42.86)	109 (66.87)
	Explicitly no systemic review of the literature is needed	16 (10,74)	1 (7.14)	17 (10.43)
Reporting standard	Refer to CONSORT or SPIRIT or both	111 (74,50)	13 (92.86)	124 (76.07)
	Do not refer to a reporting standard	38 (25,5)	1 (7.14)	39 (23.93)

Data are presented as the number of journals in the indicated category, which comply with the findings in *n* (%)

## Discussion

The primary finding of this study is that the examined PTJs and LJs rarely require the use of SRs for the justification of new trials. The majority of journals analysed refer to reporting standards such as CONSORT or SPIRIT. The CONSORT statement calls for an "adequate background" for the rationale of new clinical trials and also states that trials should “include a reference to a systematic review of previous similar trials or a note of the absence of such trials” [33].

However, as described in the introduction, a large number of studies show that SRs are rarely used to develop study rationales [13–17]. In physiotherapy and rehabilitation research, this applies to only one-third of all studies reviewed [18].

Simply referring to reporting standards such as CONSORT, without explicitly requiring reference to SRs in the author guidelines, does not appear to be sufficient to encourage authors to comply and use SRs in their study justification. Clear requirements in author guidelines, such as those used by The Lancet, may be more likely to result in compliance with the methods described.

The EBR Statement requires the justification of new research with a systematic review of the existing literature, placing new results in the context of the existing literature, and including the end-users’ perspective [9, 10, 34]. According to the EBR Statement, relevant stakeholders have different responsibilities in the research process [9]. While researchers should be able to prioritise research questions, taking into account all previous and ongoing research on the topic, and know how to find, evaluate and develop SRs, editors and journals should assess and evaluate whether the research is adequately described in the context of a SR [9]. Problems with placing current clinical research in the context of previous findings may indicate that editors and journals are not assessing the submitted papers critically enough and against the specifications of the author guidelines or their selected referrals to reporting standards like the CONSORT statement.

Although several studies show that new clinical trials are not adequately referencing SRs, multiple meta-research studies show a significant increase in the total number of SRs conducted [35–37]. Hoffmann et al. 2021 describe a 20-fold increase in SRs from 2000 to 2019 which also plays an important role in the production of research waste. According to Ioannidis, masses of unnecessary, misleading and contradictory SRs are being published which are unable to provide an accurate assessment of the current state of research, again highlighting the importance of knowing how to critically appraise SRs [35].

This difficulty is particularly important in physiotherapy, where progress in different areas of research is uneven. While in some areas like osteoarthritis research, a series of Cochrane reviews indicate that no further research on the effects of exercise is needed [5–8], there are multiple areas where there is hardly any data or high-quality studies yet [38–41].

A strength of this meta-research study is that author guidelines were systematically screened and journals were selected in a transparent manner. A limitation of this study is that only 149 PTJs were examined, so it is possible that not all journals potentially relevant to physiotherapy were captured. Also, PTJs were not included if they did not meet the cut-off of 22 publications. Thereby, journals with a low publication rate in other physiotherapy-related SCIE categories, such as ‘Rheumatology’, might not have been captured. Furthermore, the quality of the underlying publications was not determined which could induce a bias to the relevance of certain journals to physiotherapy.

## Conclusion

Based on the findings of this study, scientific medical journals do not require their authors explicitly in their author guidelines to base a new trial on an existing SR. The majority of the analysed journals refer to the CONSORT statement which requires the use of SRs for the study justification. Only journals associated with The Lancet explicitly require the use of SRs in their author guidelines. With regard to the hypothesis of this study, it can be concluded that PTJs require the use of SRs for the study justification less than LJs. The results of this work show that PTJs rely on checklists such as the CONSORT statement instead of demanding the legitimisation of new studies through a SR of the existing literature. This potentially leaves room for unethical scientific practices and should be critically considered in future research.

### Supplementary Information

**Additional file 1.** Table with full data of results.

## Data Availability

The exact results of this study are shown in the supplements.

## Abbreviations

CONSORT: Consolidated Standards of Reporting Trials
EBR: Evidence-Based Research
EQUATOR: Enhancing the Quality and Transparency Of health Research
ICMJE: International Committee of Medical Journal Editors
JCR: Journal Citation Reports
LJs: Leading scientific medical journals
PTJs: Physiotherapy-related scientific medical journals
RCT/RCTs: Randomized Controlled Trial/Randomized Controlled Trials
SCIE: Science Citation Index Expanded
SPIRIT: Standard Protocol Items: Recommendations for Interventional Trials
SR/ SRs: Systematic Review/Systematic Reviews
