# Supplementary material for: The use of systematic reviews for conducting new studies in physiotherapy research: a meta-research study comparing author guidelines of physiotherapy-related journals
Source: Syst Rev. 2024 Jan 13;13:28. doi: 10.1186/s13643-023-02427-7 (PMC10787449; doi:10.1186/s13643-023-02427-7)
Supplement: Supplementary file 1 — Additional file 1. Table with full data of results. [file 13643_2023_2427_MOESM1_ESM.docx]

Supplements

The following tables illustrate the results of this study.

The journal titles include hyperlinks to the author guideline pages of the journals. “#NV“ indicates that this information wasn’t found.

PEDro represents the number of publications indexed in the PEDro database(1). PubMed indicates how many publications were indexed in the PubMed database(2). In the JCR column, the journal’s impact factor is indicated.

The numbers in the column “rationale” indicate whether SRs are required to justify a new study. The numbers indicate 2 if it is a requirement to use SRs; 1 if an appropriate background is required; 0 if nothing regarding background is mentioned; -1 if explicitly no systemic review of the literature is needed.

The numbers in the column reporting standard indicate 1: The journal refers to CONSORT(3) or SPIRIT(4) (might be through referring to the ICMJE(5)); 0: The journal doesn’t refer to a reporting standard.

**Table S1: PTJs**

| Journal title with hyperlink | PEDro | PubMed | JCR | Publisher | WOS Category | Rationale | Reporting standard |
| --- | --- | --- | --- | --- | --- | --- | --- |
| [CLINICAL REHABILITATION](https://journals.sagepub.com/author-instructions/CRE) | 276 | 367 | 3.477 | SAGE Publications Ltd | REHABILITATION | 0 | 1 |
| [JOURNAL OF PHYSICAL THERAPY SCIENCE](https://jpts.spts.jpn.com/for_authors/instructions_authors/) | 178 | 0 | #NV | #NV | #NV | 1 | 0 |
| [INTERNATIONAL JOURNAL OF ENVIRONMENTAL RESEARCH & PUBLIC HEALTH](https://www.mdpi.com/journal/ijerph) | 167 | 89 | 3.390 | MDPI Multidisciplinary Digital Publishing Institute | PUBLIC, ENVIRONMENTAL & OCCUPATIONAL HEALTH | 0 | 1 |
| [ARCHIVES OF PHYSICAL MEDICINE AND REHABILITATION](https://www.elsevier.com/wps/find/journaldescription.cws_home/623354?generatepdf=true) | 160 | 64 | 3.966 | W.B. Saunders Ltd | REHABILITATION | -1 | 1 |
| [PLOS ONE](https://journals.plos.org/plosone/s/submission-guidelines#loc-clinical-trials) | 141 | 96 | 3.240 | Public Library of Science | #NV | 2 | 1 |
| [JOURNAL OF BODYWORK AND MOVEMENT THERAPIES](https://www.elsevier.com/journals/journal-of-bodywork-and-movement-therapies/1360-8592/guide-for-authors) | 139 | 304 | #NV | Churchill Livingstone | #NV | 0 | 1 |
| [JOURNAL OF BACK AND MUSCULOSKELETAL REHABILITATION](https://www.iospress.com/authorship-policy) | 134 | 146 | 1.398 | IOS Press | ORTHOPEDICS | 0 | 0 |
| [MEDICINE AND SCIENCE IN SPORTS AND EXERCISE](https://edmgr.ovid.com/msse/accounts/ifauth.htm) | 103 | 41 | 5.411 | Lippincott Williams and Wilkins Ltd. | SPORT SCIENCES | 0 | 0 |
| [EUROPEAN JOURNAL OF PHYSICAL AND REHABILITATION MEDICINE](https://www.minervamedica.it/en/journals/europa-medicophysica/notice-to-authors.php) | 98 | 155 | 2.874 | Edizioni Minerva Medica S.p.A. | REHABILITATION | 0 | 1 |
| [MEDICINE](http://journals.lww.com/md-journal/pages/default.aspx) | 97 | 221 | 1.889 | Elsevier BV | MEDICINE, GENERAL & INTERNAL | 0 | 1 |
| [AMERICAN JOURNAL OF PHYSICAL MEDICINE & REHABILITATION](https://edmgr.ovid.com/ajpmr/accounts/ifauth.htm) | 89 | 73 | 2.159 | Lippincott Williams and Wilkins Ltd. | REHABILITATION | 0 | 1 |
| [DISABILITY AND REHABILITATION](https://www.tandfonline.com/action/authorSubmission?show=instructions&journalCode=idre20) | 77 | 74 | 3.033 | Informa Healthcare | REHABILITATION | 0 | 1 |
| [EXPERIMENTAL GERONTOLOGY](https://www.elsevier.com/journals/experimental-gerontology/0531-5565/guide-for-authors) | 77 | 47 | 4.032 | Elsevier Inc. | #NV | -1 | 0 |
| [JOURNAL OF SPORTS MEDICINE AND PHYSICAL FITNESS](https://www.minervamedica.it/en/journals/sports-med-physical-fitness/notice-to-authors.php) | 77 | 174 | 1.637 | Edizioni Minerva Medica S.p.A. | SPORT SCIENCES | 0 | 1 |
| [SCANDINAVIAN JOURNAL OF MEDICINE & SCIENCE IN SPORTS](https://authorservices.wiley.com/ethics-guidelines/index.html#18) | 77 | 152 | 4.221 | Blackwell Munksgaard | SPORT SCIENCES | 0 | 1 |
| [COMPLEMENTARY THERAPIES IN CLINICAL PRACTICE](https://www.elsevier.com/journals/complementary-therapies-in-clinical-practice/1744-3881/guide-for-authors) | 68 | 150 | 2.446 | Churchill Livingstone | INTEGRATIVE & COMPLEMENTARY MEDICINE | 0 | 1 |
| [PHYSIOTHERAPY THEORY AND PRACTICE](http://informahealthcare.com/ptp) | 68 | 106 | 2.279 | Informa Healthcare | REHABILITATION | 0 | 0 |
| [INDIAN JOURNAL OF PHYSIOTHERAPY AND OCCUPATIONAL THERAPY](https://www.ijpot.com/articles.html) | 66 | 0 | #NV | #NV | #NV | -1 | 0 |
| [BMC MUSCULOSKELETAL DISORDERS](https://bmcmusculoskeletdisord.biomedcentral.com/submission-guidelines/conditions-of-publication) | 65 | 112 | 2.362 | BioMed Central Ltd. | ORTHOPEDICS | 0 | 1 |
| [COMPLEMENTARY THERAPIES IN MEDICINE](https://www.elsevier.com/journals/complementary-therapies-in-medicine/0965-2299/guide-for-authors) | 64 | 115 | 2.446 | Churchill Livingstone | INTEGRATIVE & COMPLEMENTARY MEDICINE | 0 | 1 |
| [JOURNAL OF CLINICAL MEDICINE](http://www.mdpi.com/journal/jcm) | 64 | 0 | 4.241 | #NV | MEDICINE, GENERAL & INTERNAL | 2 | 1 |
| [JOURNAL OF REHABILITATION MEDICINE](https://www.google.com/url?sa=t&rct=j&q=&esrc=s&source=web&cd=&ved=2ahUKEwjvzo299fT0AhXhRPEDHT4rBqUQFnoECA8QAQ&url=https%3A%2F%2Fwww.medicaljournals.se%2Fjrm%2F&usg=AOvVaw3IOE038zcq4OruiQnQCLeA) | 64 | 52 | 2.912 | 0 | REHABILITATION | 0 | 1 |
| [EVIDENCE-BASED COMPLEMENTARY AND ALTERNATIVE MEDICINE](https://www.hindawi.com/journals/ecam/guidelines/) | 62 | 0 | 2.629 | Hindawi Publishing Corporation | INTEGRATIVE & COMPLEMENTARY MEDICINE | 0 | 1 |
| [JOURNAL OF STRENGTH & CONDITIONING RESEARCH](https://www.editorialmanager.com/JSCR/default.aspx) | 62 | 238 | 3.775 | NSCA National Strength and Conditioning Association | SPORT SCIENCES | -1 | 0 |
| [SCIENTIFIC REPORTS](https://www.nature.com/srep/journal-policies/editorial-policies#clinical-trials) | 61 | 15 | 4.379 | Nature Publishing Group | #NV | 0 | 1 |
| [NEUROREHABILITATION](https://www.iospress.com/catalog/journals/neurorehabilitation) | 60 | 71 | 2.138 | IOS Press | CLINICAL NEUROLOGY | 0 | 1 |
| [BMJ OPEN](http://bmjopen.bmj.com/) | 59 | 65 | 2.692 | BMJ Publishing Group | MEDICINE, GENERAL & INTERNAL | 1 | 1 |
| [SUPPORTIVE CARE IN CANCER](https://www.springer.com/journal/520/submission-guidelines) | 58 | 88 | 3.603 | Springer Verlag | HEALTH CARE SCIENCES & SERVICES | 0 | 1 |
| [JAMA NETWORK OPEN](https://jamanetwork.com/journals/jamanetworkopen/pages/instructions-for-authors) | 56 | 9 | 8.483 | American Medical Association | #NV | 0 | 1 |
| [JOURNAL OF AGING AND PHYSICAL ACTIVITY](https://journals.humankinetics.com/view/journals/japa/japa-overview.xml?tab_body=null-10883) | 54 | 29 | 1.961 | Human Kinetics Publishers Inc. | GERIATRICS & GERONTOLOGY | -1 | 1 |
| [PHYSICAL THERAPY](https://academic.oup.com/ptj/pages/Author_Guidelines) | 54 | 33 | #NV | Oxford University Press | ORTHOPEDICS | 0 | 1 |
| [INDIAN JOURNAL OF PUBLIC HEALTH RESEARCH & DEVELOPMENT](https://ijphrd.com/articles.html) | 52 | 0 | #NV | #NV | #NV | 0 | 0 |
| [JOURNAL OF ALTERNATIVE AND COMPLEMENTARY MEDICINE](https://home.liebertpub.com/publications/journal-of-alternative-and-complementary-medicine-the/26/overview) | 52 | 117 | 2.579 | Mary Ann Liebert Inc. | INTEGRATIVE & COMPLEMENTARY MEDICINE | 0 | 1 |
| [JOURNAL OF MANIPULATIVE AND PHYSIOLOGICAL THERAPEUTICS](https://www.elsevier.com/journals/journal-of-manipulative-and-physiological-therapeutics/0161-4754/guide-for-authors) | 52 | 51 | 1.437 | Mosby Inc. | HEALTH CARE SCIENCES & SERVICES | -1 | 1 |
| [JOURNAL OF PHYSIOTHERAPY](http://www.journalofphysiotherapy.com/) | 50 | 30 | 7.000 | Australian Physiotherapy Association | ORTHOPEDICS | 0 | 1 |
| [NEUROREHABILITATION AND NEURAL REPAIR](https://journals.sagepub.com/author-instructions/NNR) | 50 | 14 | 3.919 | SAGE Publications Inc. | CLINICAL NEUROLOGY | 2 | 1 |
| [JOURNAL OF SPORT REHABILITATION](https://journals.humankinetics.com/view/journals/jsr/jsr-overview.xml?tab_body=null-10883) | 49 | 98 | 1.931 | Human Kinetics Publishers Inc. | REHABILITATION | -1 | 0 |
| [JOURNAL OF THE AMERICAN GERIATRICS SOCIETY](https://agsjournals.onlinelibrary.wiley.com/hub/journal/15325415/forauthors.html) | 49 | 26 | 5.562 | Wiley-Blackwell Publishing Ltd | GERIATRICS & GERONTOLOGY | 0 | 1 |
| [TURKISH JOURNAL OF PHYSICAL MEDICINE AND REHABILITATION](http://www.ftrdergisi.com/eng/Anasayfa=20) | 49 | 0 | 1.078 | Turkish Society of Physical Medicine and Rehabilitation | REHABILITATION | 0 | 1 |
| [JOURNAL OF MEDICAL INTERNET RESEARCH](http://www.jmir.org/) | 48 | 19 | 5.428 | Journal of medical Internet Research | HEALTH CARE SCIENCES & SERVICES | 0 | 0 |
| [JOURNAL OF PHYSICAL ACTIVITY & HEALTH](https://journals.humankinetics.com/view/journals/jpah/jpah-overview.xml) | 48 | 9 | 2.592 | #NV | #NV | 0 | 1 |
| [FRONTIERS IN PHYSIOLOGY](http://journal.frontiersin.org/journal/physiology) | 47 | 0 | 4.566 | Frontiers Media S.A. | PHYSIOLOGY | 2 | 1 |
| [LASERS IN MEDICAL SCIENCE](https://www.springer.com/journal/10103/submission-guidelines) | 47 | 89 | 3.161 | Springer London | SURGERY | 0 | 1 |
| [BMC PUBLIC HEALTH](https://bmcpublichealth.biomedcentral.com/about?gclid=EAIaIQobChMI0fjG7f709AIVBM53Ch1tXwQVEAAYASAAEgKQcfD_BwE) | 45 | 70 | 3.295 | BioMed Central Ltd. | PUBLIC, ENVIRONMENTAL & OCCUPATIONAL HEALTH | 0 | 1 |
| [BRITISH JOURNAL OF SPORTS MEDICINE](https://bjsm.bmj.com/pages/authors/) | 44 | 59 | 13.800 | BMJ Publishing Group | SPORT SCIENCES | 0 | 1 |
| [GAIT & POSTURE](https://www.elsevier.com/wps/find/journaldescription.cws_home/525442?generatepdf=true) | 44 | 39 | 2.840 | Elsevier | NEUROSCIENCES | 0 | 1 |
| [INTERNATIONAL JOURNAL OF BEHAVIORAL NUTRITION AND PHYSICAL ACTIVITY](https://ijbnpa.biomedcentral.com/) | 44 | 6 | 6.457 | BioMed Central Ltd. | NUTRITION & DIETETICS | 0 | 1 |
| [BMC GERIATRICS](https://bmcgeriatr.biomedcentral.com/submission-guidelines) | 42 | 33 | 3.921 | BioMed Central Ltd. | GERIATRICS & GERONTOLOGY | 0 | 1 |
| [JMIR MHEALTH AND UHEALTH](https://mhealth.jmir.org/author-information/instructions-for-authors) | 42 | 9 | 4.773 | Journal of medical Internet Research | HEALTH CARE SCIENCES & SERVICES | 0 | 0 |
| [PAIN MEDICINE](https://painmedicine.org.ua/) | 42 | 37 | 3.750 | VICER Publishing | ANESTHESIOLOGY | 0 | 0 |
| [CLINICAL INTERVENTIONS IN AGING](http://www.dovepress.com/clinical-interventions-in-aging-journal) | 41 | 56 | 4.458 | Dove Medical Press Ltd. | GERIATRICS & GERONTOLOGY | 0 | 0 |
| [PHYSIOTHERAPY](https://www.elsevier.com/journals/physiotherapy/0031-9406/guide-for-authors) | 41 | 88 | 3.358 | Elsevier | REHABILITATION | 0 | 1 |
| [PHYSIOTHERAPY RESEARCH INTERNATIONAL](https://onlinelibrary.wiley.com/page/journal/14712865/homepage/forauthors.html) | 41 | 68 | #NV | Wiley-Blackwell | #NV | 1 | 1 |
| [EUROPEAN JOURNAL OF APPLIED PHYSIOLOGY](https://www.springer.com/journal/421/submission-guidelines#Instructions%20for%20Authors_Types%20of%20papers) | 40 | 116 | 3.078 | Springer Verlag | PHYSIOLOGY | 0 | 1 |
| [JOURNAL OF SCIENCE AND MEDICINE IN SPORT](https://www.elsevier.com/journals/journal-of-science-and-medicine-in-sport/1440-2440/guide-for-authors) | 40 | 63 | 4.319 | Elsevier BV | SPORT SCIENCES | 0 | 1 |
| [ARTHRITIS CARE AND RESEARCH](https://onlinelibrary.wiley.com/page/journal/21514658/homepage/forauthors.html) | 39 | 2 | #NV | John Wiley and Sons Inc. | RHEUMATOLOGY | 0 | 1 |
| [BIOMED RESEARCH INTERNATIONAL](https://www.hindawi.com/journals/bmri/guidelines/) | 39 | 39 | 3.411 | Hindawi Publishing Corporation | MEDICINE, RESEARCH & EXPERIMENTAL | 0 | 1 |
| [JOURNAL OF STROKE & CEREBROVASCULAR DISEASES](https://www.elsevier.com/journals/journal-of-stroke-and-cerebrovascular-diseases/1052-3057/guide-for-authors) | 39 | 57 | 2.136 | W.B. Saunders Ltd | NEUROSCIENCES | 0 | 0 |
| [JOURNAL OF ORTHOPAEDIC AND SPORTS PHYSICAL THERAPY](https://www.jospt.org/page/authors/prepareManuscript) | 38 | 51 | #NV | Movement Science Media | #NV | 0 | 1 |
| [ARCHIVES OF GERONTOLOGY AND GERIATRICS](https://www.elsevier.com/journals/archives-of-gerontology-and-geriatrics/0167-4943/guide-for-authors) | 37 | 30 | 3.250 | Elsevier Ireland Ltd | GERIATRICS & GERONTOLOGY | -1 | 1 |
| [JOURNAL OF CLINICAL AND DIAGNOSTIC RESEARCH](https://www.jcdr.net/editorial_statement.asp) | 37 | 0 | #NV | #NV | #NV | 1 | 0 |
| [JOURNAL OF SPORTS SCIENCES](https://www.editorialmanager.com/rjsp/default.aspx) | 37 | 93 | 3.337 | Routledge | SPORT SCIENCES | 0 | 0 |
| [JOURNAL OF NEUROENGINEERING & REHABILITATION](https://www.biomedcentral.com/getpublished/editorial-policies) | 36 | 14 | 4.262 | BioMed Central Ltd. | NEUROSCIENCES | 0 | 1 |
| [MUSCULOSKELETAL SCIENCE AND PRACTICE](https://www.elsevier.com/wps/find/journaldescription.cws_home/740330?generatepdf=true) | 36 | 32 | 2.520 | Elsevier BV | REHABILITATION | 0 | 1 |
| [PILOT AND FEASIBILITY STUDIES](https://www.biomedcentral.com/getpublished/editorial-policies) | 36 | 0 | #NV | BioMed Central Ltd. | #NV | 0 | 1 |
| [RESPIRATORY CARE](http://rc.rcjournal.com/content/author-guidelines#ethics) | 36 | 30 | 2.258 | Daedalus Enterprises Inc. | CRITICAL CARE MEDICINE | 0 | 1 |
| [INTERNATIONAL JOURNAL OF REHABILITATION RESEARCH](https://edmgr.ovid.com/ijrr/accounts/ifauth.htm) | 35 | 42 | 1.479 | Lippincott Williams and Wilkins Ltd. | REHABILITATION | 0 | 1 |
| [JOURNAL OF CARDIOPULMONARY REHABILITATION AND PREVENTION](https://edmgr.ovid.com/jcrp/accounts/ifauth.htm) | 35 | 14 | 2.081 | Lippincott Williams and Wilkins Ltd. | CARDIAC & CARDIOVASCULAR SYSTEMS | 0 | 0 |
| [JOURNAL OF EXERCISE REHABILITATION](https://www.e-jer.org/authors/authors.php) | 35 | 0 | #NV | Korean Society of Exercise Rehabilitation | #NV | 1 | 0 |
| [JOURNALS OF GERONTOLOGY - SERIES A BIOLOGICAL SCIENCES AND MEDICAL SCIENCES](https://academic.oup.com/biomedgerontology/pages/General_Instructions_2) | 35 | 11 | #NV | Oxford University Press | GERIATRICS & GERONTOLOGY | 0 | 1 |
| [JOURNAL OF HAND THERAPY](https://www.elsevier.com/wps/find/journaldescription.cws_home/672751?generatepdf=true) | 34 | 34 | 1.950 | Hanley and Belfus Inc. | ORTHOPEDICS | 1 | 1 |
| [AGING CLINICAL AND EXPERIMENTAL RESEARCH](https://www.springer.com/journal/40520/submission-guidelines) | 33 | 65 | 3.636 | Springer Verlag | GERIATRICS & GERONTOLOGY | 0 | 1 |
| [JPMA. THE JOURNAL OF THE PAKISTAN MEDICAL ASSOCIATION](https://jpma.org.pk/view-instructions) | 32 | 40 | #NV | Pakistan Medical Association | MEDICINE, GENERAL & INTERNAL | 1 | 1 |
| [OBESITY](http://onlinelibrary.wiley.com/journal/10.1002/(ISSN)1930-739X) | 32 | 16 | 5.002 | Wiley-Blackwell | ENDOCRINOLOGY & METABOLISM | 0 | 0 |
| [AMERICAN JOURNAL OF SPORTS MEDICINE](https://journals.sagepub.com/author-instructions/AJS) | 30 | 25 | 6.202 | SAGE Publications Inc. | ORTHOPEDICS | 1 | 1 |
| [CLINICAL JOURNAL OF SPORT MEDICINE](https://edmgr.ovid.com/cjsm/accounts/ifauth.htm#Original) | 30 | 29 | 3.638 | Lippincott Williams and Wilkins Ltd. | ORTHOPEDICS | 0 | 0 |
| [EUROPEAN JOURNAL OF SPORT SCIENCE](https://www.tandfonline.com/action/authorSubmission?journalCode=tejs20&page=instructions) | 30 | 72 | 4.050 | Taylor and Francis Ltd. | SPORT SCIENCES | 0 | 0 |
| [FRONTIERS IN AGING NEUROSCIENCE](http://journal.frontiersin.org/journal/aging-neuroscience) | 30 | 0 | 5.750 | Frontiers Media S.A. | GERIATRICS & GERONTOLOGY | 2 | 1 |
| [INTERNATIONAL JOURNAL OF CLINICAL AND EXPERIMENTAL MEDICINE](http://www.ijcem.com/guidelines.html) | 30 | 0 | #NV | e-Century Publishing Corporation | MEDICINE, RESEARCH & EXPERIMENTAL | 0 | 0 |
| [INTERNATIONAL JOURNAL OF NURSING STUDIES](https://www.elsevier.com/wps/find/journaldescription.cws_home/266?generatepdf=true) | 30 | 20 | 5.837 | Elsevier Ltd. | NURSING | 0 | 1 |
| [BRAZILIAN JOURNAL OF PHYSICAL THERAPY](https://www.elsevier.com/journals/brazilian-journal-of-physical-therapy/1413-3555/guide-for-authors) | 29 | 31 | 3.377 | Elsevier Editora Ltda | ORTHOPEDICS | -1 | 1 |
| [INTERNATIONAL JOURNAL OF SPORTS MEDICINE](http://www.thieme.com/sportsmed) | 29 | 74 | 3.118 | Georg Thieme Verlag | SPORT SCIENCES | 0 | 1 |
| [JOURNAL OF ALZHEIMER'S DISEASE](https://www.j-alz.com/prep) | 29 | 0 | #NV | IOS Press | #NV | 0 | 0 |
| [FRONTIERS IN NEUROLOGY](http://journal.frontiersin.org/journal/neurology) | 28 | 0 | 4.003 | Frontiers Media S.A. | CLINICAL NEUROLOGY | 2 | 1 |
| [JOURNAL OF THE AMERICAN MEDICAL DIRECTORS ASSOCIATION](https://www.elsevier.com/journals/journal-of-the-american-medical-directors-association/1525-8610/guide-for-authors) | 28 | 11 | 4.669 | Elsevier Inc. | GERIATRICS & GERONTOLOGY | 1 | 0 |
| [EUROPEAN JOURNAL OF PREVENTIVE CARDIOLOGY](https://academic.oup.com/eurjpc/pages/general-instructions) | 26 | 9 | 7.804 | SAGE Publications Ltd | CARDIAC & CARDIOVASCULAR SYSTEMS | 0 | 1 |
| [HEALTH TECHNOLOGY ASSESSMENT](https://www.journalslibrary.nihr.ac.uk/information-for-authors/getting-started/editorial-review-process.htm) | 26 | 2 | 4.014 | National Co-ordinating Centre for HTA | HEALTH CARE SCIENCES & SERVICES | 0 | 1 |
| [INTERNATIONAL JOURNAL OF CARDIOLOGY](https://www.elsevier.com/wps/find/journaldescription.cws_home/506041?generatepdf=true) | 26 | 30 | 4.164 | Elsevier Ireland Ltd | CARDIAC & CARDIOVASCULAR SYSTEMS | 1 | 1 |
| [OSTEOARTHRITIS AND CARTILAGE](http://www.elsevier.com/wps/find/journaldescription.cws_home/623055/description) | 26 | 12 | 6.576 | W.B. Saunders Ltd | #NV | 1 | 1 |
| [PAIN](http://journals.lww.com/pain/) | 26 | 13 | 6.961 | Lippincott Williams and Wilkins Ltd. | ANESTHESIOLOGY | 0 | 1 |
| [PHYSICAL THERAPY IN SPORT](https://www.elsevier.com/journals/physical-therapy-in-sport/1466-853X/guide-for-authors) | 26 | 52 | 2.365 | Elsevier BV | REHABILITATION | 0 | 1 |
| [IRANIAN JOURNAL OF OBSTETRICS, GYNECOLOGY AND INFERTILITY](http://ijogi.mums.ac.ir/) | 25 | 0 | #NV | Mashhad University of Medical Sciences | #NV | 0 | 0 |
| [JOURNAL OF GERIATRIC PHYSICAL THERAPY](https://edmgr.ovid.com/jgpt/accounts/ifauth.htm) | 25 | 0 | 3.381 | Lippincott Williams and Wilkins Ltd. | GERIATRICS & GERONTOLOGY | 0 | 1 |
| [NUTRIENTS](https://www.mdpi.com/journal/nutrients/instructions) | 25 | 180 | 5.717 | MDPI Multidisciplinary Digital Publishing Institute | NUTRITION & DIETETICS | 2 | 1 |
| [ACUPUNCTURE IN MEDICINE](http://aim.bmj.com/) | 24 | 20 | 2.267 | ? | INTEGRATIVE & COMPLEMENTARY MEDICINE | 0 | 1 |
| [MEDICAL SCIENCE MONITOR](https://www.medscimonit.com/instructions) | 24 | 30 | 2.649 | International Scientific Literature Inc. | MEDICINE, RESEARCH & EXPERIMENTAL | 0 | 1 |
| [NEUROUROLOGY AND URODYNAMICS](http://onlinelibrary.wiley.com/journal/10.1002/%28ISSN%291520-6777) | 24 | 26 | 2.696 | Wiley-Liss Inc. | UROLOGY & NEPHROLOGY | 0 | 1 |
| [PM&R](http://www.pmrjournal.org/) | 24 | 31 | 2.298 | #NV | REHABILITATION | 0 | 1 |
| [TOPICS IN STROKE REHABILITATION](https://www.tandfonline.com/action/authorSubmission?show=instructions&journalCode=ytsr20) | 24 | 25 | 2.119 | Taylor and Francis Ltd. | REHABILITATION | 0 | 0 |
| [GERIATRICS & GERONTOLOGY INTERNATIONAL](https://onlinelibrary.wiley.com/page/journal/14470594/homepage/forauthors.html) | 23 | 37 | 2.730 | Wiley-Blackwell | GERIATRICS & GERONTOLOGY | 1 | 0 |
| [TRIALS](https://trialsjournal.biomedcentral.com/submission-guidelines/preparing-your-manuscript/research) | 23 | 132 | 2.279 | BioMed Central Ltd. | MEDICINE, RESEARCH & EXPERIMENTAL | 0 | 1 |
| [APPLIED PHYSIOLOGY, NUTRITION AND METABOLISM](https://cdnsciencepub.com/journal/apnm/authors#guidelines) | 22 | 96 | #NV | National Research Council of Canada | NUTRITION & DIETETICS | 1 | 1 |
| [EUROPEAN JOURNAL OF CARDIOVASCULAR NURSING](https://academic.oup.com/eurjcn/pages/general-instructions) | 22 | 5 | 3.908 | SAGE Publications Inc. | CARDIAC & CARDIOVASCULAR SYSTEMS | 2 | 1 |
| [EUROPEAN JOURNAL OF INTEGRATIVE MEDICINE](https://www.elsevier.com/journals/european-journal-of-integrative-medicine/1876-3820/guide-for-authors) | 22 | 0 | 1.314 | Elsevier GmbH | INTEGRATIVE & COMPLEMENTARY MEDICINE | -1 | 1 |
| [GAMES FOR HEALTH JOURNAL](https://home.liebertpub.com/publications/games-for-health-journal/588/for-authors) | 22 | 14 | 3.204 | Mary Ann Liebert Inc. | #NV | 0 | 1 |
| [INTEGRATIVE CANCER THERAPIES](https://journals.sagepub.com/author-instructions/ICT) | 22 | 8 | 3.279 | SAGE Publications Inc. | INTEGRATIVE & COMPLEMENTARY MEDICINE | 1 | 1 |
| [INTERNATIONAL JOURNAL OF THERAPY AND REHABILITATION](https://www.magonlinelibrary.com/page/authors/submissions) | 22 | 0 | #NV | Mark Allen Publishing Ltd. | #NV | 0 | 1 |
| [KNEE SURGERY, SPORTS TRAUMATOLOGY, ARTHROSCOPY](https://www.springer.com/journal/167/submission-guidelines) | 22 | 60 | #NV | Springer Verlag | ORTHOPEDICS | 0 | 1 |
| [THE JOURNAL OF MANUAL & MANIPULATIVE THERAPY](https://www.tandfonline.com/action/authorSubmission?show=instructions&journalCode=yjmt20#checklist) | 22 | 12 | #NV | Maney Publishing | #NV | 0 | 1 |
| [THORAX](http://thorax.bmj.com/) | 22 | 4 | 9.139 | BMJ Publishing Group | RESPIRATORY SYSTEM | 2 | 1 |
| [ANNALS OF PHYSICAL AND REHABILITATION MEDICINE](https://www.elsevier.com/wps/find/journaldescription.cws_home/718644?generatepdf=true) | 20 | 29 | 4.919 | Elsevier Masson | REHABILITATION | 1 | 1 |
| [BMC CANCER](https://bmccancer.biomedcentral.com/about) | 20 | 44 | 4.430 | BioMed Central Ltd. | ONCOLOGY | 0 | 1 |
| [BREAST CANCER RESEARCH AND TREATMENT](https://www.springer.com/journal/10549/submission-guidelines) | 20 | 24 | 4.872 | Springer New York | ONCOLOGY | 0 | 1 |
| [EUROPEAN JOURNAL OF PAIN](https://onlinelibrary.wiley.com/page/journal/15322149/homepage/forauthors.html) | 20 | 23 | 3.931 | Wiley-Blackwell | ANESTHESIOLOGY | 0 | 1 |
| [JOURNAL OF ATHLETIC TRAINING](https://meridian.allenpress.com/DocumentLibrary/NATA/2020_JAT_Authors'_Guide.pdf) | 20 | 25 | 2.860 | National Athletic Trainers' Association Inc. | SPORT SCIENCES | 1 | 1 |
| [JOURNAL OF SPORTS SCIENCE & MEDICINE](https://www.jssm.org/newauthors.php) | 20 | 23 | 2.988 | Department of Sports Medicine, Medical Faculty of Uludag University | SPORT SCIENCES | 0 | 0 |
| [SPINE](https://edmgr.ovid.com/spine/accounts/ifauth.htm) | 20 | 31 | 3.468 | Lippincott Williams and Wilkins Ltd. | CLINICAL NEUROLOGY | 0 | 0 |
| [CLINICAL BIOMECHANICS](https://www.elsevier.com/journals/clinical-biomechanics/0268-0033/guide-for-authors) | 19 | 16 | 2.063 | Elsevier Ltd. | ORTHOPEDICS | 0 | 1 |
| [INTERNATIONAL JOURNAL OF CHRONIC OBSTRUCTIVE PULMONARY DISEASE](https://www.dovepress.com/journal-editor-international-journal-of-chronic-obstructive-pulmonary-disease-eic6) | 19 | 31 | 3.355 | COPD Foundation | RESPIRATORY SYSTEM | 0 | 1 |
| [INTERNATIONAL UROGYNECOLOGY JOURNAL](https://www.springer.com/journal/192/submission-guidelines) | 19 | 38 | 2.894 | Springer London | OBSTETRICS & GYNECOLOGY | -1 | 1 |
| [MULTIPLE SCLEROSIS AND RELATED DISORDERS](https://www.elsevier.com/journals/multiple-sclerosis-and-related-disorders/2211-0348/guide-for-authors) | 19 | 34 | 4.339 | Elsevier | CLINICAL NEUROLOGY | -1 | 1 |
| [ALTERNATIVE THERAPIES IN HEALTH AND MEDICINE](http://www.alternative-therapies.com/) | 18 | 33 | 1.305 | InnoVision Communications | INTEGRATIVE & COMPLEMENTARY MEDICINE | 0 | 1 |
| [OSTEOPOROSIS INTERNATIONAL](https://www.springer.com/journal/198/submission-guidelines) | 17 | 26 | 4.507 | Springer London | #NV | -1 | 0 |
| [BMC COMPLEMENTARY AND ALTERNATIVE MEDICINE now BMC COMPLEMENTARY MEDICINE AND THERAPIES](https://bmccomplementmedtherapies.biomedcentral.com/about) | 16 | 36 | 3.659 | BioMed Central Ltd. | INTEGRATIVE & COMPLEMENTARY MEDICINE | 0 | 1 |
| [CLINICAL RHEUMATOLOGY](https://www.springer.com/journal/10067/submission-guidelines) | 16 | 37 | 2.980 | Springer London | #NV | 0 | 1 |
| [SPINAL CORD](https://www.nature.com/documents/scsandc-gta.pdf) | 14 | 36 | 2.772 | Nature Publishing Group | CLINICAL NEUROLOGY | 0 | 1 |
| [SCIENCE AND SPORTS](https://www.elsevier.com/journals/science-and-sports/0765-1597/guide-for-authors) | 13 | 0 | 0.789 | Elsevier Masson | SPORT SCIENCES | 1 | 0 |
| [DEVELOPMENTAL NEUROREHABILITATION](http://informahealthcare.com/pdr) | 12 | 26 | 2.308 | Informa Healthcare | CLINICAL NEUROLOGY | 0 | 0 |
| [INTERNATIONAL JOURNAL OF BIOMETEOROLOGY](https://www.springer.com/journal/484/submission-guidelines) | 12 | 24 | 3.787 | Springer New York | PHYSIOLOGY | 1 | 1 |
| [JOURNAL OF BONE AND JOINT SURGERY. AMERICAN VOLUME](https://journals.lww.com/jbjsjournal/Pages/Instructions-for-Authors.aspx) | 12 | 26 | 5.284 | Lippincott Williams and Wilkins Ltd. | ORTHOPEDICS | 0 | 1 |
| [JOURNAL OF SHOULDER AND ELBOW SURGERY](https://www.elsevier.com/journals/journal-of-shoulder-and-elbow-surgery/1058-2746/guide-for-authors) | 12 | 24 | 3.019 | Mosby Inc. | ORTHOPEDICS | -1 | 1 |
| [JOURNAL OF ADVANCED NURSING](https://onlinelibrary.wiley.com/page/journal/13652648/homepage/forauthors.html) | 11 | 22 | 3.187 | Wiley-Blackwell Publishing Ltd | NURSING | 0 | 1 |
| [ORTHOPAEDIC JOURNAL OF SPORTS MEDICINE](https://journals.sagepub.com/author-instructions/OJS) | 11 | 0 | 2.727 | SAGE Publications Inc. | ORTHOPEDICS | 0 | 1 |
| [ISOKINETICS AND EXERCISE SCIENCE](https://www.iospress.com/catalog/journals/isokinetics-and-exercise-science) | 10 | 0 | 0.519 | IOS Press | ORTHOPEDICS | 0 | 1 |
| [PAIN PHYSICIAN](http://www.painphysicianjournal.com/) | 10 | 27 | 4.965 | Association of Pain Management Anesthesiologists | ANESTHESIOLOGY | 0 | 1 |
| [RESEARCH IN SPORTS MEDICINE](https://www.tandfonline.com/action/authorSubmission?show=instructions&journalCode=gspm20) | 10 | 23 | 4.674 | Taylor and Francis Ltd. | SPORT SCIENCES | 0 | 0 |
| [JOURNAL OF ORTHOPAEDIC SURGERY AND RESEARCH](https://josr-online.biomedcentral.com/about) | 9 | 14 | 2.359 | BioMed Central Ltd. | ORTHOPEDICS | 0 | 1 |
| [JOURNAL OF TELEMEDICINE AND TELECARE](https://journals.sagepub.com/author-instructions/JTT) | 9 | 26 | 6.184 | SAGE Publications Ltd | HEALTH CARE SCIENCES & SERVICES | 0 | 1 |
| [OCCUPATIONAL THERAPY INTERNATIONAL](https://www.hindawi.com/journals/oti/guidelines/) | 9 | 13 | 1.448 | Hindawi Limited | REHABILITATION | 0 | 1 |
| [ARCHIVES OF ORTHOPAEDIC AND TRAUMA SURGERY](https://www.springer.com/journal/402/submission-guidelines) | 8 | 10 | 3.067 | Springer Verlag | ORTHOPEDICS | 0 | 1 |
| [BMC NEUROLOGY](https://bmcneurol.biomedcentral.com/about) | 8 | 40 | 2.474 | BioMed Central Ltd. | CLINICAL NEUROLOGY | 0 | 1 |
| [FOOT AND ANKLE INTERNATIONAL](https://us.sagepub.com/en-us/nam/foot-ankle-international/journal202135#submission-guidelines) | 8 | 10 | #NV | SAGE Publications Inc. | ORTHOPEDICS | 1 | 0 |
| [NEUROLOGICAL SCIENCES](https://www.springer.com/journal/10072/submission-guidelines) | 8 | 25 | 3.307 | Springer-Verlag Italia | CLINICAL NEUROLOGY | 0 | 1 |
| [JOURNAL OF ARTHROPLASTY](https://www.elsevier.com/wps/find/journaldescription.cws_home/623141?generatepdf=true) | 7 | 25 | 4.757 | Churchill Livingstone | ORTHOPEDICS | 1 | 1 |
| [INTERNATIONAL JOURNAL OF SPORTS PHYSIOLOGY AND PERFORMANCE](https://journals.humankinetics.com/view/journals/ijspp/ijspp-overview.xml?tab_body=null-10883) | 6 | 58 | 4.010 | Human Kinetics Publishers Inc. | PHYSIOLOGY | 1 | 0 |
| [INTERNATIONAL JOURNAL OF SPORT NUTRITION AND EXERCISE METABOLISM](https://journals.humankinetics.com/view/journals/ijsnem/ijsnem-overview.xml?tab_body=null-10883) | 3 | 24 | 4.599 | Human Kinetics Publishers Inc. | NUTRITION & DIETETICS | 0 | 0 |
| [CLINICAL PHYSIOLOGY AND FUNCTIONAL IMAGING](https://onlinelibrary.wiley.com/page/journal/1475097x/homepage/forauthors.html) | 2 | 35 | 2.273 | Wiley-Blackwell Publishing Ltd | PHYSIOLOGY | -1 | 0 |
| [EUROPEAN JOURNAL OF NUTRITION](https://www.springer.com/journal/394/submission-guidelines) | 1 | 32 | 5.614 | D. Steinkopff-Verlag | NUTRITION & DIETETICS | 0 | 1 |
| [NEUROMODULATION : JOURNAL OF THE INTERNATIONAL NEUROMODULATION SOCIETY](https://www.elsevier.com/wps/find/journaldescription.cws_home/747975?generatepdf=true) | 1 | 53 | 4.722 | Wiley-Blackwell Publishing Ltd | CLINICAL NEUROLOGY | -1 | 0 |

**Table S2: LJs:**

|  | PEDro | PubMed | JCR | Publisher | WOS Category | Rationale | Reporting standard |
| --- | --- | --- | --- | --- | --- | --- | --- |
| [NEW ENGLAND JOURNAL OF MEDICINE](https://www.nejm.org/author-center/new-manuscripts) | 0 | 0 | 91.245 | Massachussetts Medical Society | MEDICINE, GENERAL & INTERNAL | 0 | 1 |
| [LANCET](https://www.thelancet.com/pb-assets/Lancet/authors/tl-info-for-authors.pdf) | 10 | 0 | 79.321 | Elsevier | MEDICINE, GENERAL & INTERNAL | 2 | 1 |
| [JAMA](https://jamanetwork.com/journals/jamanetworkopen/pages/instructions-for-authors) | 34 | 9 | 56.272 | Jama Network | MEDICINE, GENERAL & INTERNAL | 0 | 1 |
| [NATURE MEDICINE](https://www.nature.com/nm/submission-guidelines) | 0 | 0 | 53.440 | Nature Publishing Group | MEDICINE, RESEARCH & EXPERIMENTAL | 0 | 1 |
| [WORLD PSYCHIATRY](https://onlinelibrary.wiley.com/page/journal/20515545/homepage/productinformation.html) | 1 | 0 | 49.548 | Wiley-Blackwell | PSYCHIATRY | 0 | 0 |
| [JOURNAL OF CLINICAL ONCOLOGY](https://ascopubs.org/jco/authors/peer-review-process) | 6 | 0 | 44.544 | American Society of Clinical Oncology | ONCOLOGY | 1 | 1 |
| [LANCET ONCOLOGY](https://www.thelancet.com/pb-assets/Lancet/authors/tlo-info-for-authors.pdf) | 0 | 0 | 41.316 | Elsevier | ONCOLOGY | 2 | 1 |
| [BMJ-BRITISH MEDICAL JOURNAL](https://www.bmj.com/about-bmj/resources-authors/article-types) | 21 | 0 | 39.890 | BMJ | MEDICINE, GENERAL & INTERNAL | 1 | 1 |
| [CANCER DISCOVERY](https://aacrjournals.org/content/authors/editorial-policies) | 0 | 0 | 39.397 | American Association for Cancer Research Inc. | ONCOLOGY | 0 | 1 |
| [ANNALS OF ONCOLOGY](https://www.elsevier.com/wps/find/journaldescription.cws_home/747533?generatepdf=true) | 2 | 0 | 32.976 | Elsevier | ONCOLOGY | 0 | 1 |
| [LANCET DIABETES & ENDOCRINOLOGY](https://els-jbs-prod-cdn.jbs.elsevierhealth.com/pb/assets/raw/Lancet/authors/tlde-info-for-authors.pdf) | 0 | 0 | 32.069 | Elsevier | ENDOCRINOLOGY & METABOLISM | 2 | 1 |
| [JAMA ONCOLOGY](https://jamanetwork.com/journals/jamaoncology/pages/instructions-for-authors) | 4 | 7 | 31.777 | American Medical Association | ONCOLOGY | 1 | 1 |
| [IMMUNITY](https://www.cell.com/immunity/authors) | 0 | 0 | 31.745 | Cell Press | IMMUNOLOGY | -1 | 1 |
| [LANCET RESPIRATORY MEDICINE](https://www.thelancet.com/pb-assets/Lancet/authors/tlrm-info-for-authors.pdf) | 0 | 0 | 30.700 | Elsevier | CRITICAL CARE MEDICINE | 2 | 1 |

References

1. PEDro. PEDro homepage [Internet]. 2021 [cited 2023 Apr 10]. Available from: https://pedro.org.au

2. PubMed. PubMed homepage [Internet]. 2021 [cited 2023 Apr 10]. Available from: https://pubmed.ncbi.nlm.nih.gov

3. Schulz KF, Altman DG, Moher D, for the CONSORT Group. CONSORT 2010 Statement: Updated Guidelines for Reporting Parallel Group Randomised Trials. PLoS Med. 2010 Mar 24;7(3):e1000251.

4. Chan AW, Tetzlaff JM, Altman DG, Laupacis A, Gøtzsche PC, Krleža-Jerić K, et al. SPIRIT 2013 Statement: Defining Standard Protocol Items for Clinical Trials. Ann Intern Med. 2013 Feb 5;158(3):200.

5. ICMJE [Internet]. 2021 [cited 2021 Feb 1]. Available from: https://www.icmje.org
